# Investigation of the Relationship between Degradation of the Coating of Gas Turbine Blades and Its Surface Color

**DOI:** 10.3390/ma14247843

**Published:** 2021-12-18

**Authors:** Mariusz Bogdan, Józef Błachnio, Artur Kułaszka, Dariusz Zasada

**Affiliations:** 1 Department of Mechanical Engineering, Bialystok Technical University, 45 Wiejska Street, 15-333 Białystok, Poland; 2 Air Force Institute of Technology, 6 Księcia Bolesława Street, 01-494 Warszawa, Poland; jozef.blachnio@itwl.pl (J.B.); artur.kulaszka@itwl.pl (A.K.); 3 Faculty of Advanced Technologies and Chemistry, Military University of Technology, 2 Kaliskiego Street, 00-908 Warszawa, Poland; dariusz.zasada@wat.edu.pl

**Keywords:** gas turbine, blade, coating, degradation, surface color

## Abstract

This article presents issues concerning the relationship between the degradation of the coating of gas turbine blades and changes in the color of its surface. Conclusions were preceded by the determination of parameters characterizing changes in the technical condition of protective coatings made based on a metallographic examination that defined the morphological modifications of the microstructure of the coating, chemical composition of oxides, and roughness parameters. It has been shown that an increased operating time causes parameters that characterize the condition of the blades to deteriorate significantly. Results of material tests were compared with those of blade surface color analyses performed using a videoscope. Image data were represented in two color models, i.e., RGB and L*a*b* with significant differences being observed between parameters in both representations. The study results demonstrated a relationship between the coating degradation degree and changes in the color of the blade’s surface. Among others, this approach may be used as a tool to assess the condition of turbine blades as well as entire gas turbines.

## 1. Introduction

**Figure 1:**
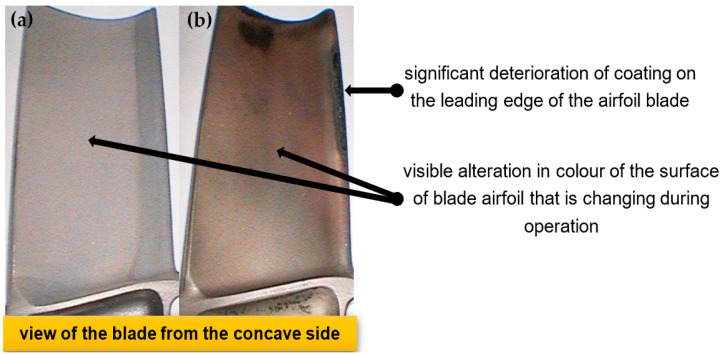
Exemplary blade images: (**a**) benchmark blade; (**b**) turbine blade leading edge damaged due to overheating.

Gas turbines used in jet engines are exposed to great thermal and mechanical loads. They are distinguished by their efficiency, which depends on the temperature of exhaust gases at the turbine inlet. It should be noted that over time, excessive temperature concerning operating temperature can lead to the degradation of the blade’s structure and thus to turbine failure [1,2,3,4,5,6]. One external symptom of blade degradation is a change of its surface color [7,8,9] (Figure 1a,b), which is a diagnostic signal indicating alteration of its technical condition. The most common method of monitoring blades uses visual optoelectronic observation where the color of the blade’s surface is recorded. This technique allows for non-invasive monitoring of the technical condition (degradation) of blades through the use of a videoscope with the initial reliability assessment (improvement of technical condition) being performed by comparing obtained images of the blade’s surface with a picture of the surface of a standard blade [5,8]. However, this type of non-invasive monitoring requires personnel who possess advanced skills, its results are more difficult to interpret, and, in comparison to invasive methods, it provides limited credibility [10,11]. For these reasons, it becomes necessary to subject blades to a metallographic examination that provides a lot more information regarding changes within their structure but excludes them from further use. To avoid such an outcome, the authors suggest a new approach for assessing the technical condition of blades. This method involves a non-invasive assessment of the blade’s surface color, whose outcome is then correlated to the results of a test of the microstructure of the blade’s surface. The use of a computer-assisted expert method as part of this evaluation will provide appropriate levels of credibility [12,13]. Furthermore, the proposed approach allows for a non-invasive indirect assessment of the technical condition of operational blades without having to remove them from the turbine and deconstructing them, thus extending their service life or decommissioning them before they cause engine failure.

Blades are often made of nickel-based alloys, which are characterized by good microstructure stability as well as good mechanical and thermal properties [14,15,16]. However, these alloys are stable only to a certain average temperature, and when it is exceeded, their desired mechanical properties start to decline [17,18,19]. To prevent blades from reaching this temperature, heat-resistant coatings exhibiting resistance to high-temperature corrosion, low thermal conductivity, and high structural stability [20,21,22] as well as possessing internal cooling systems [23,24,25] are frequently used. Blade operating conditions force designers to use nickel alloys that are resistant to high heat and high-temperature creep, high-temperature sulfuric corrosion, thermal wear, overheating, and especially creep [14,15,26,27]. Various phenomena that can deteriorate the condition of blades can occur within the operational process. Erosion, for example, causes pitting, which results in the formation of oxidation between the coating and the blade’s surface, causing the delamination of the heat-resistant layer resulting in fatigue cracks [26,27]. High-temperature sulfur corrosion, which penetrates erosion cracks, results in the delamination of the heat-resistant coating and the corrosion of the superalloy [28,29,30]. It also contributes to the formation of some degraded zones with locally deteriorated thermal and mechanical properties. This kind of damage may cause, for example, the overheating and fatigue cracking of the blade’s superalloy [31,32,33]. Excessively high temperatures exceeding nominal temperatures which persist for a long time as well as the occurrence of high centrifugal forces during rotation can result in superalloy creep [18,34,35]. Additionally, it is essential that the temperature of the exhaust gases does not fluctuate while the turbine engine is at a constant rotational speed and that it is evenly distributed around its circumference [36]. Fluctuations of as well as a lack of even distribution of temperature around the turbine’s circumference result in the formation of zones where the temperature of exhaust gases is excessive. It should also be mentioned that the total (accumulated) temperature of exhaust gases that pass the leading edge of the blade is significantly higher than that measured at the back of the turbine. The value of the total temperature depends on the static temperature, the square of the velocity of exhaust gas flow, enthalpy, and the gas constant of these exhaust gases [37].

Section 2.1 defines the subject of study and identifies the scope of both material tests as well as the conducted color analysis. The realization of metallographic tests and the procedure for the calibration and correction of recorded image data have been described (Section 2.2). Characteristics of the method for color analysis (comparison of properties of RGB and L*a*b* color spaces) are supplied in the context of a color description of a blade’s leading edge. The completed material studies (Section 3.1) are aimed at the determination of the state of the coating/blades within the area of the leading edge and concern a set of five blades: a new, unused, blade and four blades (internally air-cooled using a channeled construction) with various lengths of exploitation. The research intentions to identify changes caused by the exploitation of these blades under complex/demanding environmental conditions and concern, on the one hand, the obtaining of information relevant from the perspective of recording of images or the state of surface degradation (among others: chemical composition, including those caused through the impact of high-temperature exhaust gasses, the thickness of oxide layers, or surface roughness) and on the other hand, microstructural changes related to the coatings themselves (such as coating thickness and the impact of high-temperature exhaust gasses). Section 3.2 details color analyses conducted on recorded images that were performed through color analyses based on sequencing within the RGB and L*a*b* color spaces. Both Section 3.2, as well as the Conclusions section, describe possibilities for arriving at diagnostic conclusions concerning the technical state of the considered blades on the basis of their surface color. The authors of the article tried to present a wide range of both material tests as well as color analyses of the imaged blades’ surfaces. Material tests, as well as color analyses, were performed at specific points, areas surrounding these points, and at the section (fragment) of the leading edge of the blade. This allowed the study of and reaching conclusions about not only specific points but also the entire selected area of the blade’s surface.

The proposed approach significantly expands possibilities connected with the non-destructive appraisal of gas turbine blades especially in the context of assessing their technical state without the need for their disassembly. The assessment of their condition may be performed through the use of a sight glass device (a videoscope)—image recording was done on real specimens. In practical applications, this solution will provide a credible method (one excluding the human factor often utilized in visual assessments) for the gauging of the degradation state of gas turbine blades based on changes to its surface color and, at the same time, it may become a tool that during periodic or inter-repair equipment reviews allows a particular blade to remain in service or disqualify it from it, thus weighing in on the safe operation of the entire turbine and the possible extension of exploitation of its blades.

## 2. Materials and Methods

### 2.1. Characteristics of the Test Object

The study considered rotor blades of a gas turbine of a jet engine operated under varying conditions. The blades were removed from the turbine after a previous assessment of their technical condition using a visual method utilizing an optoelectronic device. The blades possess a multi-channel structure, allowing them to be cooled internally with air, since the maximum static temperature of exhaust gases behind the turbine can reach 860 °C. The considered blades were made of a nickel–cobalt superalloy (Table 1) having a monocrystalline structure. Using the condensation–diffusion method, the entire surface of the turbine blade had been coated with a coating bearing a Russian name SDP-2 and having a thickness of 40–80 μm. However, the blade’s leading edge was additionally coated with a coating bearing a Russian name WSDP-16 consisting of Ni, Al, Cr, Y, and having a thickness of 75–100 μm. These coatings protect the blade from gas corrosion within 1000–1100 °C temperature ranges.

**Figure 2:**
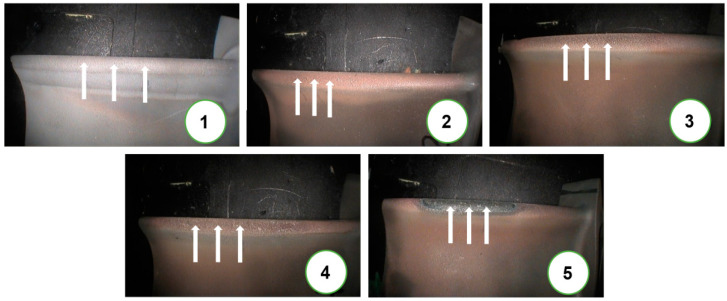
Turbine blade leading edges imaged using a MENTOR VISUAL IQ videoscope with an adopted classification of their technical condition and an indication of places where tests of the coating were performed. (1) blade No. 1; (2) blade No. 2; (3) blade No. 3; (4) blade No. 4; (5) blade No. 5.

An exemplary set of test objects in the form of leading edges of a turbine rotor’s blade has been shown in Figure 2. They have been arranged in order starting from a new blade to one that is showing the most deterioration (the images are marked with numbers from 1 to 5, with 1 indicating the new blade). The technical condition of the above-mentioned test objects was determined on the basis of a visual assessment based on the expert knowledge of the authors of the present article gained through numerous years of experience. Places where material tests were performed have been marked with arrows. The location of these tests is caused by the fact that in a vast majority of cases, degradation of the coating starts on the surface of the leading edge. Provided examples confirm this fact, since the selected set of blades are more deteriorated at the leading edge than in the remaining parts of their surfaces. The state of their degradation reflects the technical condition of particular blades. It should be mentioned that the conducted analysis focused solely on determining changes in the technical condition of the leading edge of the exemplary set of blades.

Indicated test sites of material tests and areas of color analysis associated with them (Figure 2) differ slightly for each blade (marked from 1 to 5). Blades that have undergone exploitation, blades 2 through 5, as well as the unused blade No. 1 were installed onto different turbine jet engines with an afterburner of the same type. During exploitation, their degrading did not progress at the same rate, which is a consequence of varying tasks performed by fighter aircraft. The use of and duration of afterburners during the operation of the engine were an important factor that significantly impacted the loads to which the blades were subjected and through that their level of degradation. For this reason, the diagnostic process assessing the serviceability of blades considers not only the number of hours they had been in use but primarily the state of their surface. Engine diagnostic tests are carried out through the utilization of optoelectronic-supported visual methods. The exploitation time of considered blades within their overhaul life was expressed through percentages: No. 1—0%, No. 2—59%, No. 3—81%, No. 4—92%, No. 5—98%.

### 2.2. Metallographic Examination of Blade Leading-Edge Coatings

After spark erosion cutting, structural test samples were mechanically ground along the leading edge of an abrasive paper with a decreasing granulation of 100–4000 µm and then polished using a diamond slurry with granulation of 3 µm. A comprehensive test of the structure and morphology of a blade’s leading edge surface, as well as an observation of changes in the coating through the use of a high-resolution Quanta 3D FEG (SEM/FIB) scanning electron microscope system equipped with an integrated EDS/WDS/EBSD system (EDS energy dispersive X-ray detector, WDS wavelength dispersive X-ray detector, and EBSD electron backscatter diffraction analysis system), were performed. The Quanta 3D FEG scanning electron microscope system guarantees high-resolution images of excellent quality (including SE—secondary electrons and BSE—backscattered electrons). The Quanta 3D FEG scanning electron microscope system works within a specimen chamber and under variable conditions including high vacuum. It is equipped with a very large measurement camera that makes it possible to observe the surface of the entire element or assembly without having to cut them into smaller elements.

The evaluation of the 2D geometrical structure was carried out at the leading edge zone of the blade, using a PGM-1C profilometer with a BS 1000-10-1 WAT head. To determine the surface profile, its roughness parameter (Ra) was determined as the arithmetic mean elevation of the profile. Measurements of coating thickness and the resulting oxide layers on their surfaces, taken previously with the Quanta scanning microscope system, were done utilizing a Nikon MA 200 inverted materials microscope equipped with a CCD camera and a Nikon NIS-BR.

Phase analysis of the leading edge of considered blades was performed using a Seifert XRD 3003 TT X-ray diffractometer (Cu lamp, 2θ angle ranging from 20 to 120°, exposure time at measuring point 3 s, progression 0.02°).

### 2.3. Examination of Color Images of Blade Leading Edge Surfaces

The examination was conducted through the use of a visual method employing a Mentor ViQ Video Probe videoscope. Table 2 presents the videoscope’s most significant characteristics concerning the examination that was performed.

**Figure 3:**
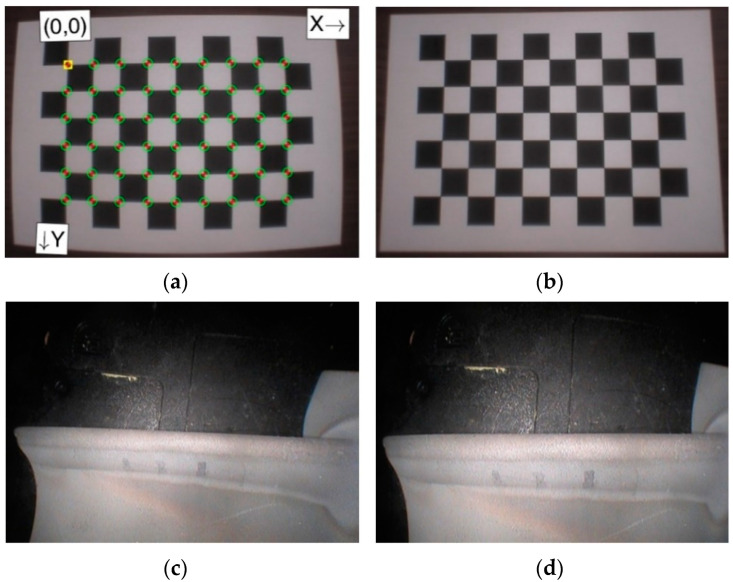
Example of a calibration pattern (**a**) and the same pattern after correction of its distortions (**b**) original blade image No. 1 (**c**) and the same blade after image correction (**d**).

Before the initiation of the tests, the videoscope was calibrated. The calibration meant to remove distortions within the optical probe was done in a Matlab environment. In this approach, the calibration algorithm for the pinhole camera was selected. The calibration process enables the determination of parameters that define the relationship between the basic system and the system related to the camera as well as optical probe and perspective transformation parameters. Parameters established through the calibration procedure of the videoscope were useful in determining the extrinsic parameters related to the displacement and rotation of the camera system compared to the system related to the image being observed as well as the intrinsic parameters that define the camera’s optical and electrical properties. Furthermore, apart from the focal length, radial and tangential distortions related to the optical system were also modeled. The actual coordinates of the camera’s image center, which was not situated in the geometrical center of the converter matrix, was also determined. A grid consisting of 10 columns and seven rows of alternating black and white fields with nominal dimensions of 24 × 24 mm (Figure 3a) was adopted as the calibration standard. At different positions and distances between the inspection camera of the Mentor Visual iQ videoscope and the test object, similar to those applied in actual inspections, a total of 51 reference images were recorded at a resolution of 1280 × 960 pixels (96 dpi) and 24-bit color depth. The calibration process allowed, to a certain extent, a reduction of distortions in original images (Figure 3b,d). Thanks to this and through the use of a slight image rotation (Figure 3a,c), a straight line of the leading edge was obtained in the resulting images (Figure 3d).

**Figure 4:**
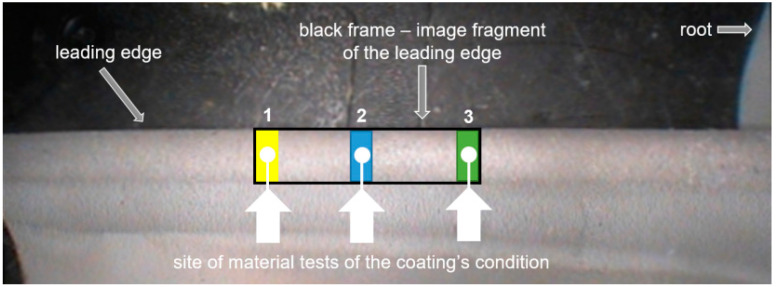
Image fragment of the surface of blade No. 1 after distortion correction and with an indication of test sites where coloring and coating’s condition was examined.

Thanks to these measures, it became possible to isolate areas (fragments) of leading edge’s images that were optimal in terms of size. The diagram shown in Figure 4 depicts the isolated fragment of the blade’s leading edge image.

The surface of the selected section (black frame) is limited by points 1 and 3 that represent places/areas, where material tests examining the condition of the coating were performed. Point 2 is located in the middle, that is, between point 1 (the most distant point from the blade root) and point 3 (the closest point to the blade root). Rectangular yellow, blue, and green frames mark areas surrounding material and test sites (image sections are 65 × 30 pixels in size). The technical condition of the coating is indicated with arrows and related white circles. The color analysis covered both entire leading edge fragments (black frame) and local smaller areas related to the areas of coating condition tests (yellow, blue, and green rectangles).

Table 3 presents images of fragments of blade edges (within the black frame) of blades 1 to 5. Depicted surfaces represent areas of different sizes and are all the same width but have varying lengths (value expressed in pixels). Three local surfaces, described in Figure 4 (size 65 × 30 pixels, surface 1—yellow rectangle, surface 2—blue rectangle, surface 3—green rectangle), were isolated (surrounding points 1, 2, 3) from each presented fragment of a leading edge.

The color analysis aimed to determine the average color for different representations of the image data. Two color spaces RGB and CIE L*a*b* were considered. Selected spaces have different descriptions and meanings, and their channels (R, G, B, or L*, a*, b* components) may independently constitute a set of parameters characterizing changes in the color of tested surfaces and describe differently surfaces of blades under varying technical conditions. In the context of blade diagnostics and the utilization of both color spaces for purposes related to describing the color of a blade’s leading edge surface, it is necessary to summarize some of their features [38,39,40]:

- RGB space is an additive model where the resultant color is the sum of component colors. The RGB color model is based on the human perception of colors. The color components are red, green, and blue. For a given device, every RGB component has a different spectral characteristic. Features include color saturation ranging from 0 to 255 (256 values) with some examples being red = (255, 0, 0), yellow = (255, 255, 0), white = (255, 255, 255), black = (0, 0, 0), segment (0, 0, 0)–(255, 255, 255)—shades of gray. The advantages of this method are that all intermediate colors can be represented by a linear combination of primary colors (which is convenient and quick to calculate). Disadvantages include the perceptual heterogeneity, i.e., weak correlations between the perceived difference of two colors and their Euclidean distance in the RGB cube, lack of intuitiveness in using the R, G, and B components in determining the color, and problems connected to color visualization relying on knowledge of RGB components. Additionally, the RGB model is sensitive to changes in the level of scene/object illumination, which may affect component values. Due to these disadvantages in many applications, it becomes necessary for an RGB image to be converted to another color space exhibiting better properties.
- The L*a*b* model is based on the theory of opposing colors where colors are perceived as a combination of red and blue or green and yellow. Individual components represent L*—brightness (0–100), a*—position between red and green (−128–128), and b*—position between blue and yellow (−128–128). One advantage of this model is that color comparison is easy. Differences between two colors are determined similarly to the distance between points within a 3D Euclidean space. The CIE L*a*b* model is a device-independent model.

## 3. Results

### 3.1. Results of the Metallographic Examination of the Blade Leading Edge Coating

The purpose of material tests was to determine the relationship between the surface coating color and blade serviceability. These tests were useful in determining the extent to which exploited blades differed in their technical condition from the new blade (coating). A description of material tests presented in Section 3.1. has been formulated as a characterization of coating modification throughout the progression of operation time.

**Figure 5:**
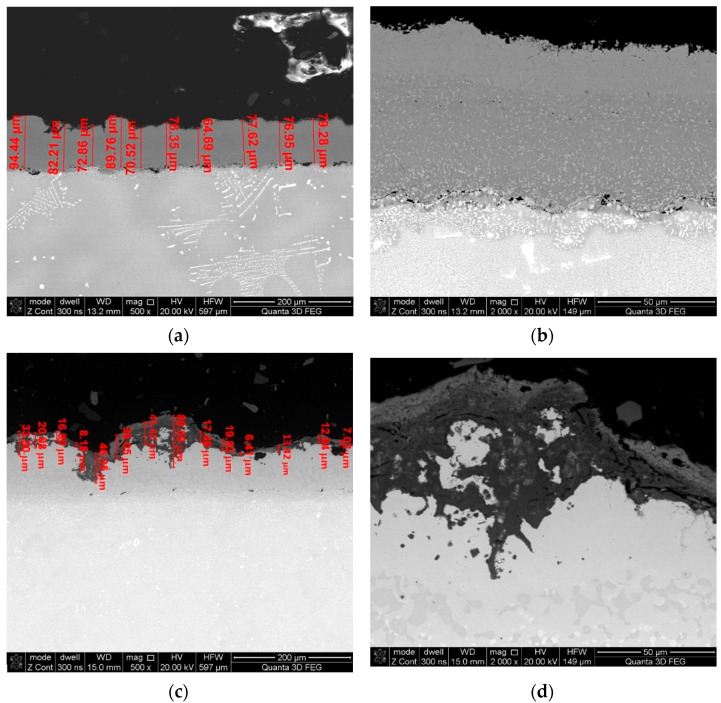
Thickness measurements: (**a**,**b**) Nz blade coating; (**c**,**d**) scale/oxides—SEM/BSE at various magnifications.

**Figure 6:**
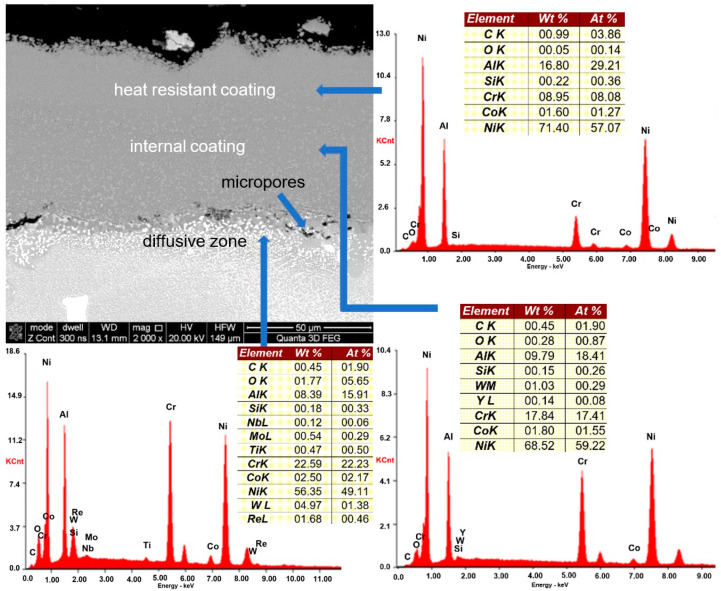
Microstructure and chemical composition of blade No. 1 coating.

Measurements of coating thickness as well as that of oxides that form on and within the coatings were conducted in three areas (marked in Figure 2) by taking 30–50 measurements of each (Figure 5). Based on microscope observations, it has been concluded that the blade was covered with a diffusive heat-resistant coating (Figure 6) consisting of a nickel aluminide matrix (intermetallic phase NiAl-β) enriched with chromium, cobalt, and yttrium. The thickness of the coating on the external surface of the blade near the leading edge was approximately 90–100 μm.

Additionally, it has been observed that the coating covering the blade’s leading edge consists of two layers: an external layer responsible for thermal resistance having an aluminum content of approximately 29% and a thickness of about 40 μm and an inner layer responsible for transferring strain containing approximately 24% Al and having a thickness of about 50 μm. The increased content of aluminum within the zone that is expected to be exposed to the highest temperatures is supposed to facilitate the formation of protective aluminum or chromium oxide layers that are created during engine operation [41,42]. Al_2_O_3_ and Cr_2_O_3_ [43] form as a result of oxygen diffusion into the coating as well as the core diffusion of aluminum and chromium. Scales of this type exhibit better protective properties than those forming on materials classified as chromia formers and therefore can be used when temperatures exceed 1000 °C. One example of such a material is the NiAl-β intermetallic phase. Furthermore, the addition of yttrium is meant to prevent porosity caused by diffusive processes occurring during operation as well as increase the adhesion of oxides [28,44,45].

**Figure 7:**
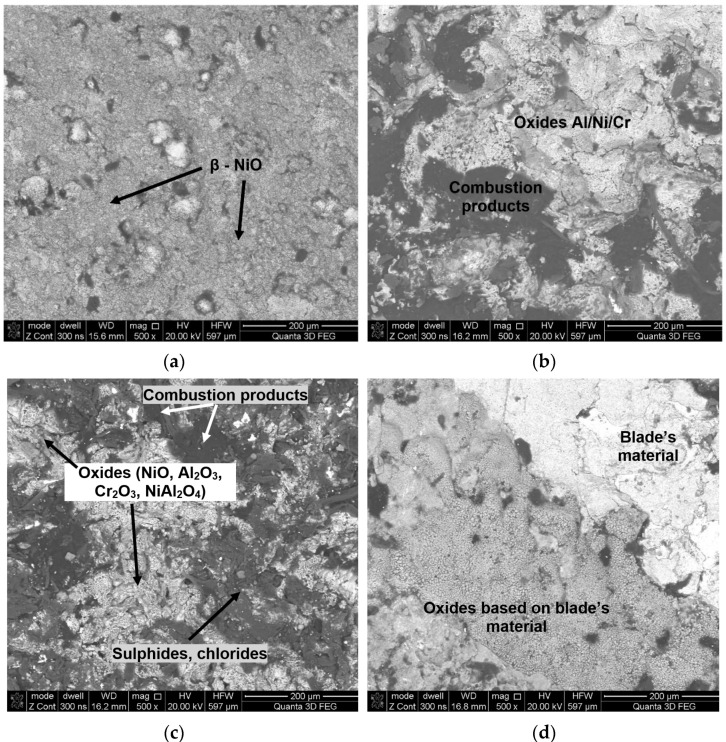
The morphology of the leading edge surface of individual BSE blades: (**a**) No. 1; (**b**) No. 2; (**c**) No. 4; (**d**) No. 5.

**Figure 8:**
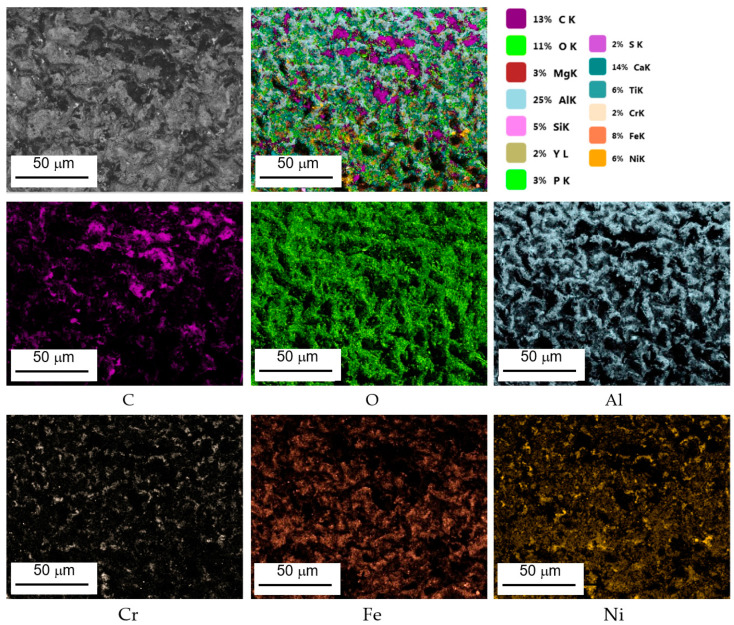
Exemplary surface distribution of selected elements, area 1 on blade No. 3.

**Figure 9:**
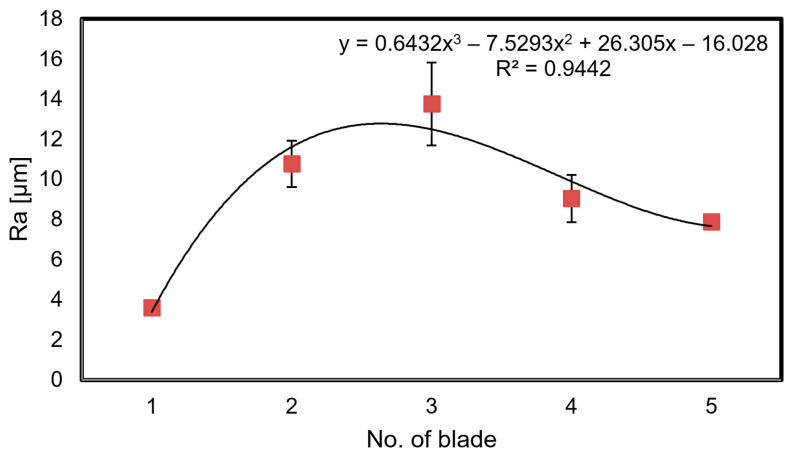
The arithmetic mean elevation of the Ra profile of surface roughness.

**Figure 10:**
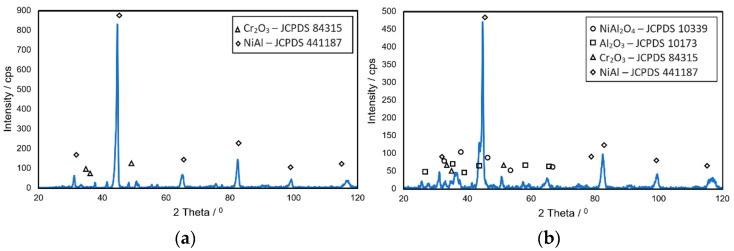
Diffractograms of blade surfaces: (**a**) new (No. 1); (**b**) exploited (No. 4).

During engine operation, the morphology of scrutinized blade surfaces has undergone adversely changes in respect to its microstructure, chemical content, phase, and roughness (Figure 7, Figure 8, Figure 9 and Figure 10). Local erosive losses and burn-through in the heat-resistant coating (Figure 7b–d), as well as the formation of scale (composed of an external layer of aluminum oxide, nickel, chromium, and fuel combustion products), have been found. Furthermore, areas significantly enriched with carbon, sulfur, and chlorine, the presence of which is directly related to components of aviation fuel or air, were observed on surfaces of tested blades. In this case, the most undesirable component of aviation fuel is sulfur, either in its free or its bound forms (e.g., sulfides, disulfides, hydrogen sulfide, and others). It has been found that the maximum content of total sulfur in fuels for turbine engines may fluctuate from 0.1% to 0.4%, while that of mercaptans ranges from 0.001% to 0.005%. Another source of contamination is polluted air or air that has not been sufficiently purified. This may be the medium through which sodium chloride, especially in seaside areas, may come in contact with turbines of aircraft engines.

Based on performed point and line analyses as well as on the mapping of chemical composition (Figure 8) in individual areas varying considerably in terms of shades of gray (BSE), potential high-temperature corrosion products had been identified (Figure 7b–d). Furthermore, point analyses performed concerning chemical composition have shown that the occurrence of products of partial or complete fuel combustion is possible in areas enriched in sulfur and chlorine [29,39] such as SO_2_, SO_3_, H_2_S, etc. This results in the formation of sulfides corresponding to the material of which the coating is made. On the other hand, in the case of blade No. 5 where the coating had completely degraded, the only oxides that were still present on the surface were ones whose chemical composition was based on the blade’s parent material. The presence of sulfur seen during operation causes the occurrence of high-temperature corrosion which, in turn, causes cracking and eventually delamination of the coating. Another element that has been reliably confirmed in tests of surfaces of blades exploited in jet engines performed by the authors is sodium. The presence of sodium sulfate (Na_2_SO_4_) at a level of several parts per million (ppm) is enough to cause extensive corrosive damages at high temperatures. Additionally, the presence of these contaminants significantly accelerates high-temperature corrosion. The above-mentioned sodium sulfate or sulfur are by-products of fuel combustion where they may exist as naturally occurring impurities.

Moreover, it was found that the roughness parameters, including Ra parameter (Figure 9), increase with the operation time. However, this may also be directly related to the increased presence of various types of oxides (e.g., Al_2_O_3_, NiO), fuel combustion products, and the formation of sulfides, chlorides, nitrides, etc. on the surface of the analyzed blades. It can also be related to an increase in the morphological heterogeneity and scale thickness. During initial operation, depending on the operating parameters, the scale thickens, causing a clear increase in the value of parameter Ra (blades 2 and 3 in Figure 9). The observed scale consists mainly of oxides formed on the basis of elements of which the coating is made (Al, Cr, Co, Ni—Figure 7b). As a result of the high diffusion of oxygen caused by the high temperature of exhaust gasses, the elements making up the surface coating react with the introduced oxygen to form various types of oxides.

At first, the oxides form an aluminum oxide layer that protects the inner surface of the coating against further oxidation. For example, Al_2_O_3_ oxide has a morphologically heterogeneous structure and a tendency to layer unevenly in the initial stage of operation, increasing roughness. Among other things, it has also been discovered that the most common components making up the scale appearing on the surface of blades are NiO, Al_2_O_3_, and NiAl_2_O_4_ oxides (Figure 10). Moreover, it has been shown that the observed increase in surface roughness of blades was higher than that seen in the initial state (Figure 9), and for blade No. 3, for example, it was three times higher.

**Figure 11:**
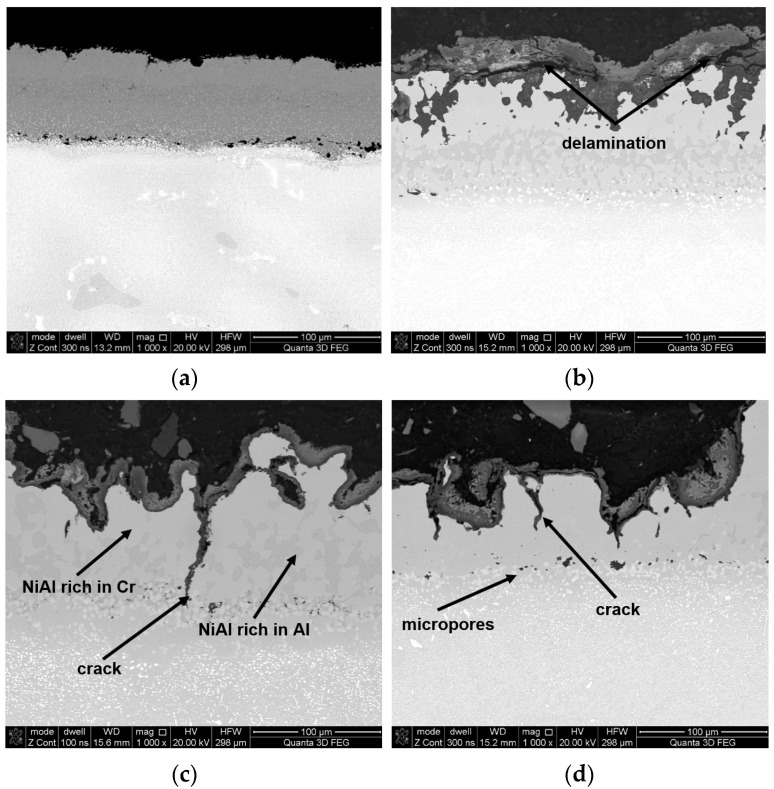

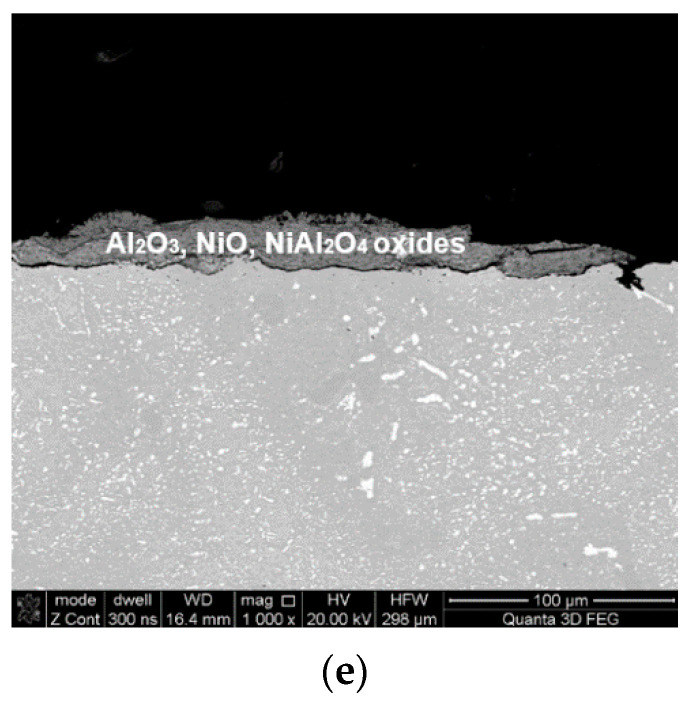
Microstructure of the surface in the vicinity of the leading edge of particular BSE blades: (**a**) No. 1; (**b**) No. 2; (**c**) No. 3; (**d**) No. 4; (**e**) No. 5.

**Figure 12:**
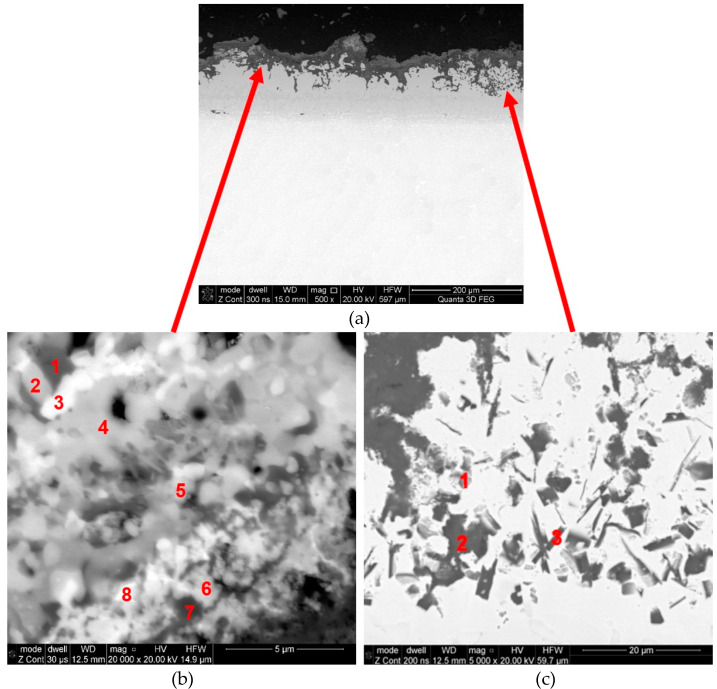
The exemplary scales with marked numbers of analyses in the region of particular phases—blade No. 2: (**a**) general view; (**b**) external coating; (**c**) internal coating.

The high-temperature impact of exhaust gases causes not only changes in the morphological and chemical composition on the surface of the exploited blades but also microstructural changes within the coatings themselves (Figure 11 and Figure 12).

Among others, it has also been discovered that as a result of the high-temperature interaction of oxygen and exhaust gases as well as diffusion processes taking place inside the coating, especially within the inner coating (Figure 6), there is a gradual unification of its chemical composition. Noted structural changes are manifested, first of all, in the redistribution of aluminum and chromium, which leads to a progressive disappearance of the separated areas of nickel aluminide enriched in aluminum or chromium (Figure 11c). In the final stage, the observed changes lead to the formation of a homogeneous coating based on the NiAl phase (compare Figure 11c with Figure 11d).

**Figure 13:**
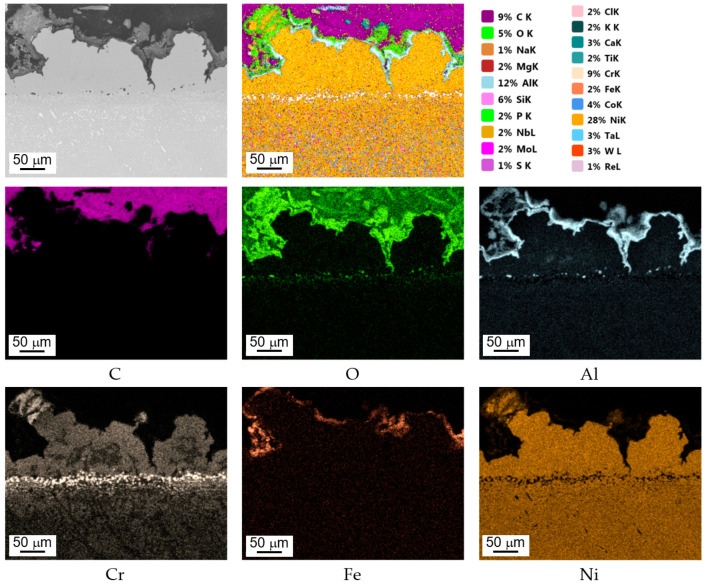
Exemplary surface distribution of selected elements cross-section, area 1 on blade No. 3.

Observed structural changes within the coating caused, among others, by the diffusion of oxygen into the coating, diffusion of the aluminum inside the core, and the diffusion of MCrAlY alloy components outside the core are accompanied by the formation of microporosity (Kirkendall effect). This process caused by the chemical impact as well as the high temperature of exhaust gases results in the formation of various types of oxides and nitrides on the surfaces of blade coatings (Figure 11, Figure 12 and Figure 13 and Table 4). It has also been discovered that as a result of high-temperature corrosion, microporosity and cracks, often running down to the parent material, appear in the inner coating (Figure 6). The occurrence of high-temperature corrosion in the above-mentioned blades is confirmed by the presence of sulfur-containing areas characterized by a specific shape (Figure 12c, Table 5). Thus, during operation, oxide and nitride layers formed on blade surfaces, and their delamination, along with microporosity, are conducive to the formation of cracks in heat-resistant coatings (Figure 12c).

**Figure 14:**
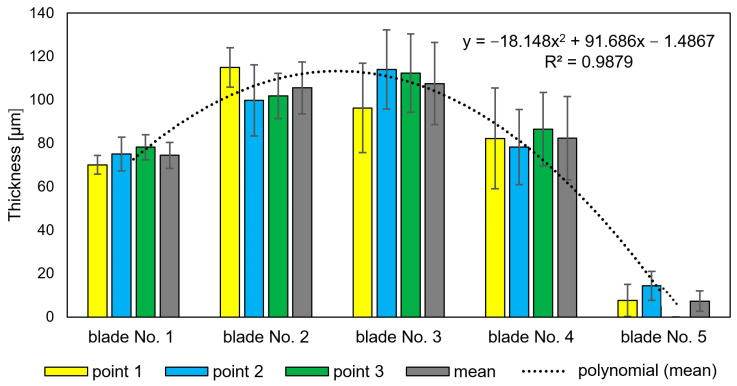
Thickness measurements of coating along with oxides.

**Figure 15:**
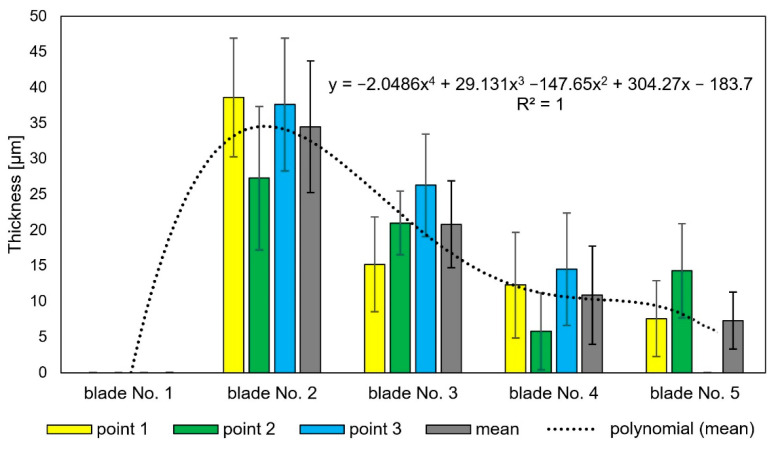
Oxide thickness measurements.

It has also been observed that as the time of operation progresses, the thickness of the coating can be seen to increase up to as much as 110 mm (blades No. 2 and 3—Figure 14). This is related to the previously described deposits of aluminum, chromium, and nickel oxides that occurred as a scale that is highly heterogeneous as well as porous on blade surfaces. These characteristics make it prone to flaking, cracking, and exfoliation. In extreme cases, these oxides penetrate the coating and react with the blade’s superalloy (Figure 11c,d), resulting in its total degradation (Figure 11e). Processes of delamination and erosion, as well as vibrations of the blade during operation, promote the flaking of these oxide layers, which leads to a reduction of the coatings’ as well as the oxide layers’ thickness (Figure 15).

### 3.2. Results of Color Analysis of Images of Blades’ Leading Edge Surfaces in Selected Zones

**Figure 16:**
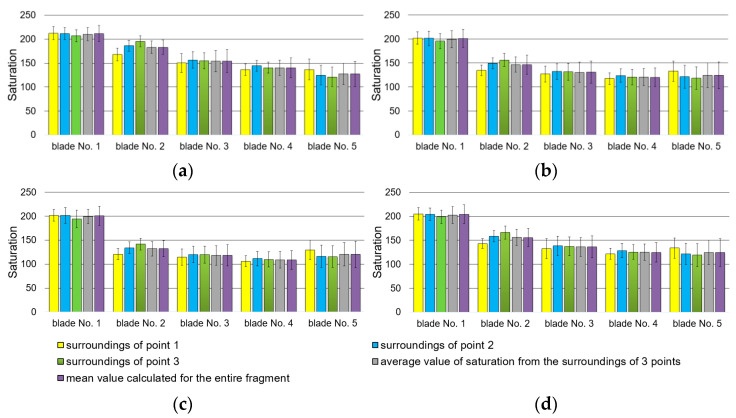
Results of surface color analysis in RGB space: (**a**) R component; (**b**) G component; (**c**) B component; (**d**) shades of gray.

**Figure 17:**
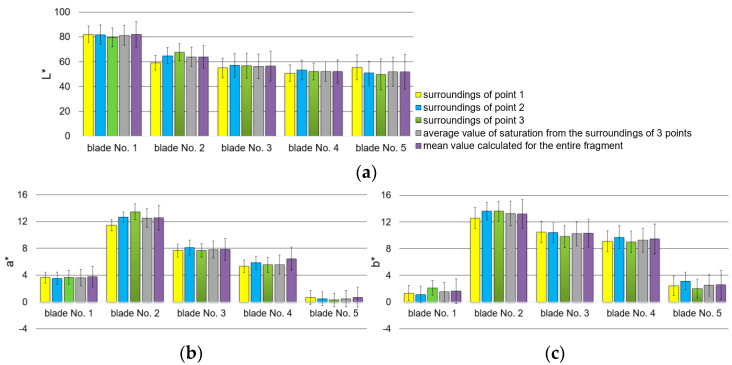
Color analysis of the surface of the leading edge fragments in L*a*b* color space, components: (**a**) L*; (**b**) a*; (**c**) b*.

Fragments of blade surfaces located at their leading edge (Figure 4) were subjected to color analysis using two color spaces, RGB and L*a*b* (Figure 16 and Figure 17).

On the basis of results presented in Figure 16, it has been ascertained that the diversification of the technical condition of blades 1 to 5 is shown in Figure 17a. Component R assumes different values of content/saturation of component R (red), especially near points 2 and 3. However, it should be mentioned that designated test sites, or locations surrounding points 1, 2, and 3, vary for individual blades. As for the average of the three areas surrounding the points (the value of the gray bar), the R component also shows color changes occurring on the surface (it is possible to determine a monotonically decreasing linear function or an approximation can be made using a 2nd-degree polynomial). The same situation applies to entire surface fragments (purple bars). It has also been noted that when it comes to blades 3 and 5, particular mean values have higher standard deviation than in other cases. These larger data differences are related to their heterogeneous surface color. The highest value of standard deviation has been recorded in relation to blade number 5 and is related to micro areas whose color is close to white (local reflections). Based on the obtained results, it has also been concluded that only in respect to blade No. 2, the surroundings of points 1, 2, and 3 show a greater differentiation of the mean value, proving the occurrence of a slight color variation within those three areas. It can also be concluded that shades of gray (Figure 16d) as well as components G and B (Figure 16b,c) do not reflect the nature of the change in surface color because blades 4 and 5 exhibit similar values of components R and B as well as those reflecting shades of gray.

The results of the color analysis presented in Figure 17 in space L*a*b* are characterized by a significant diversification of the mean value, especially concerning components a* and b* for all blades. However, L* component values (brightness) allow for some unreliability, similarly to values of components G and B within the RGB color space, because blades 4 and 5 have similar mean values: those determined from the three designated areas (surrounding measurement points) as well as those established for the entire fragment.

**Figure 18:**
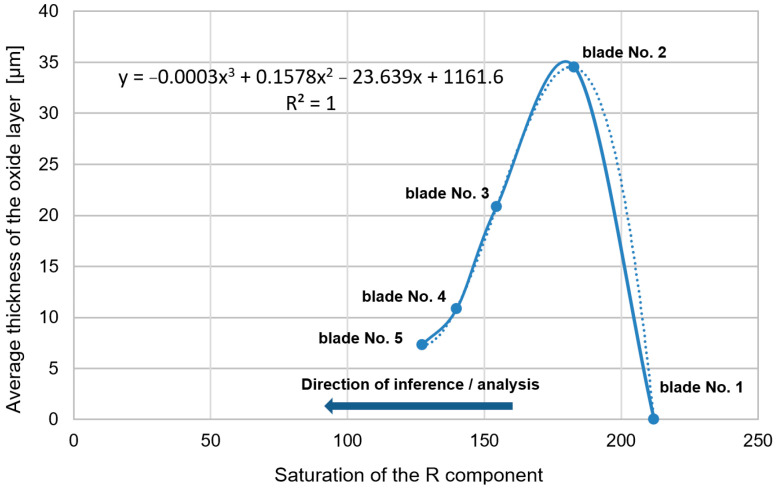
Changes in the saturation value of component R determining the thickness of the layer of oxides on the coating (a non-linear relationship).

Figure 18 presents an example of a diagram picturing changes in the saturation values of component R (Figure 16a, purple bars, mean values of R determined for entire fragments of blade leading edge surface—Table 3) qualifying changes in the thickness of the layer of oxides. As the saturation value of component R (starting from a thickness of approximately 212) decreases, the thickness of the oxide layer increases until the value of component R saturation (≈183) and a further decrease in the saturation value of component R signifies a reduction in the thickness of the layer of oxides. Therefore, changes in the saturation value of component R may provide information regarding the thickness of the layer of oxides while at the same time indicating whether the thickness of that layer will increase or decrease along with the time of operation.

## 4. Conclusions

During their operation, the technical condition of gas turbine blades undergoes changes connected to the degradation of their protective insulation coating. The greatest impact of the thermodynamic factor (exhaust gases) resulting from the operation of the gas turbine as well as the design of the blade profile occurs on the leading edge of the blade’s aerofoil. Due to its susceptibility to the influence of various adverse effects, including higher temperature (beyond the range of nominal operating temperatures), this zone is the site of most extensive damages. One of the factors that adds to the impact of increased temperature on blade surfaces involves carbon deposits that form on injectors, causing thermal damage to those injectors as well as resulting in incorrect fuel atomization and localized overheating of blades.

Considerations were preceded by a determination of the technical condition of coatings of internally operated multi-channel air-cooled blades. Material tests were useful in determining the degree of coating degradation for a set of blades marked from 1 to 5 (Section 3.1). Selected parameters, including changes in the thickness of a blade’s coating as well as that of the oxide scale occurring on it, its microstructure and chemical composition, as well as its roughness, were used to determine the scope of deterioration (degradation) of the insulating protective layer in relation to that of an unused blade (blade No. 1) or one whose operating time was not long (blade No. 2). These tests allowed the determination of changes occurring on the surface of tested blades along with the progression of their time of operation.

The second aspect addressed in the article was a color analysis of the leading edge surface of the same, previously mentioned, set of blades. Blade surface color, established through the use of a videoscope, is the result of numerous factors including the properties of the surface such as its roughness, chemical composition, and nature and direction of incident light falling on it, as well as properties of the utilized optoelectronic system (recording of the luminous flux reflected from the surface and its processing–forming). Due to the design of their inspection probe, sight glass devices have some limitations, and the calibration of the Mentor videoscope, allowing a reduction of distortions of original images (Figure 3), was necessary before initiation of the analysis. It also facilitated the selection of areas (fragments) of leading edge images that were optimal in terms of size (Figure 4). When it comes to the interpretation of color analysis results, from among the numerous options (classification possibilities) of describing changes in the color of the leading edge surface concerning the varying condition (material tests) of that surface, consideration of only one component of either the RGB model or the L*a*b* model seems to be most appropriate. In the authors’ opinion, since it makes it possible to refer to a certain specific range of averaged color information of a surface in relation to its technical condition (blades 1 through 5), component a* is the most appropriate (Figure 17b). Individual blades considered in the study are characterized by a certain range of values of the variability of component a* that define each blade, and that of blade 1 (new blade) does not coincide with those of the other blades (No. 2, 3, or 4). It is also possible to show a certain correlation of mean values of component a* with values of the arithmetic mean elevation of profile Ra (Figure 9) or coating thickness (Figure 14) for blades 1 through 5.

The above-described approach concerning color analysis and its interpretation can additionally be expanded to also include component R (Figure 16a), which will provide even more reliable diagnostic information. The means of component R can be approximated using a second-degree polynomial or its variability range—the mean with standard deviation and in respect to a*, the range of data variability—a mean with standard deviation. This approach allows for the identification of the technical condition of the blade on the basis of two different premises. Component R describes the share of red in image data and is susceptible to changes in the levels of illumination, while component a* expresses the position of the data between red and green. Additionally, component a* is not device-specific, and component L* reflects information concerning brightness.

Color changes result directly and indirectly from the structural, morphological, chemical, and phase changes within the surface layer of the coating and the parent material of the blade after complete degradation or its exfoliation. As a result of long-term operation or improper fuel combustion, corrosive and erosive wear occurs very rapidly, generating local cracks or burns in the heat-resistant coating, causing a local reduction in its thickness. Additionally, these places also become sites where scale consisting of an outer layer of nickel oxide and an inner layer of aluminum oxide forms. The high-temperature impact of exhaust gases on the analyzed coating areas also results in a redistribution of alloy components, leading to the formation of a two-phase structure of nickel aluminide and chromium-enriched nickel aluminides. Strong processes of carbide precipitation, as well as expansion and clearly noticeable changes in the morphology of the γ’ phase, occur under the coating’s surface. Other factors, such as the composition of the fuel and air purity, also have an impact on the sulfur, chloride, and combustion products formed on the surface of analyzed coatings. All of these processes contribute to the degradation of the selected areas of blades’ surfaces, which, in turn, has a direct impact on the color of these places.

An extended time of operation and, to a greater extent, the declining efficiency of fuel injectors resulting in the build-up of carbon deposits, causes increasingly incorrect fuel atomization and local extreme overheating of the blades’ coating. These processes make it much more conducive to degradation, the aggregation of a highly porous scale, and the depletion of aluminum in areas covered by such scale. Increasing amounts of scale contribute to elevated stressing of areas subjected to such corrosion and, consequently, lead to the formation of cracks within the coating itself as well as between the scale and the aluminide coating. Subsequent stages of corrosion and erosion lead to the degradation of areas of affected coating and increase the roughness or waviness of its outer surface. The last stage of wear involves the complete destruction of these coatings, and, in extreme cases, even their delamination. It has been observed that the scale that formed on the blade’s parent material is more uniform in terms of its geometrical structure. Furthermore, it has also been noted that the main components of the scale forming on the surface of analyzed blades are NiO, Al_2_O_3_, and NiAl_2_O_4_ oxides.

Summarizing, it has been concluded that since it explains changes in their serviceability through the relationship between surface structure degradation and its color in reflected light, the proposed solution, in addition to other, existing non-destructive methods [8,9,13,45,46] for the assessment of turbine blades, is of particular importance.

## Figures and Tables

**Table 1:** The basic chemical composition of the ZhS 32 alloy of blades, SEM-EDX (% of mass).

Al	Cr	Co	Nb	Mo	Ta	W	Re	Ni
6.3	5.5	10.6	1.4	1.3	0.5	9.1	3.0	62.0

**Table 2:** The examination was conducted using a visual method employing a Mentor ViQ Video Probe videoscope.

Features/Parameters	Description
optical probe	diameter 6.1 mm
image converter	color CCD Camera SUPER HEAD 1/10″
lighting	white LED
recorded image	1280 × 960 pixels (resolution 96 dpi)
3D phase measurement, 3D stereo measurement, comparative measurement	additional options

**Table 3:** Fragment sections of the leading edge of five blades.

Blade No.	Surface Image of the Leading Edge	Dimensions of Fragment Images of the Leading Edge (Width × Height) in Pixels
1	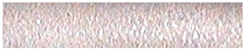	376 × 65
2	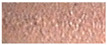	167 × 65
3	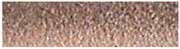	257 × 65
4	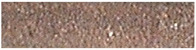	277 × 65
5	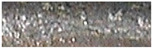	214 × 65

**Table 4:** Microanalysis of the chemical composition in marked zones according to Figure 12a (mass content wt %).

Zone	C	O	Mg	Al	Si	Y	P	Ca	Ti	Cr	Fe	Ni
1	7.96	32.64	1.20	18.47	16.92	0.00	1.43	14.49	2.61	0,12	3.88	0.29
2	4.95	31.55	4.01	18.63	9.83	0.00	0.56	11.60	3.75	0.21	13.64	1.27
3	5.46	32.19	2.35	9.35	4.22	0.32	0.39	18.99	15.70	0.16	9.78	1.09
4	5.56	30.23	4.59	17.52	6.27	0.11	0.44	10.75	3.36	0.33	18.77	2.07
5	2.40	33.47	2.18	23.08	4.55	0.24	0.80	12.35	8.29	0.46	10.63	1.55
6	1.95	30.61	3.26	17.26	5.91	3.19	0.46	8.29	3.61	1.89	14.49	9.08
7	4.10	29.99	1.60	19.09	15.27	0.77	0.43	12.02	2.12	1.52	8.28	4.81
8	3.33	30.61	2.28	14.64	14.56	0.37	0.77	16.93	3.51	0.60	11.74	1.65

**Table 5:** Microanalysis of the chemical composition in marked zones according to Figure 12b (mass content wt %).

Zone	N	O	Al	Si	S	Cr	Co	Ni
1	7.61	1.44	34.05	0.30	0.13	11.89	1.52	43.06
2	---	30.90	61.19	---	---	4.58	---	3.34
3	12.93	0.78	62.21	---	---	5.61	0.57	17.90

## Data Availability

Not applicable.
